# A field survey using LAMP assay for detection of *Schistosoma mansoni* in a low-transmission area of schistosomiasis in Umbuzeiro, Brazil: Assessment in human and snail samples

**DOI:** 10.1371/journal.pntd.0006314

**Published:** 2018-03-13

**Authors:** Javier Gandasegui, Pedro Fernández-Soto, Antonio Muro, Constança Simões Barbosa, Fabio Lopes de Melo, Rodrigo Loyo, Elainne Christine de Souza Gomes

**Affiliations:** 1 Infectious and Tropical Diseases Research Group (e-INTRO), Biomedical Research Institute of Salamanca-Research Centre for Tropical Diseases at the University of Salamanca (IBSAL-CIETUS), Faculty of Pharmacy, University of Salamanca, Salamanca, Spain; 2 Schistosomiasis Laboratory and Reference Service, Department of Parasitology, Aggeu Magalhães Institute, Fiocruz - Ministry of Health (MoH), Recife, Pernambuco, Brazil; University of California Berkeley, UNITED STATES

## Abstract

**Background:**

In Brazil, schistosomiasis is a parasitic disease of public health relevance, mainly in poor areas where *Schistosoma mansoni* is the only human species encountered and *Biomphalaria straminea* is one of the intermediate host snails. A nested-PCR based on a specific mitochondrial *S*. *mansoni* minisatellite DNA region has been successfully developed and applied as a reference method in Brazil for *S*. *mansoni* detection, mainly in host snails for epidemiological studies. The amplification efficiency of LAMP is known to be higher than PCR. The present work aimed to assess the utility of our previously described SmMIT-LAMP assay for *S*. *mansoni* detection in human stool and snail samples in a low-transmission area of schistosomiasis in the municipality of Umbuzeiro, Paraíba State, Northeast Region of Brazil.

**Methodology/Principal findings:**

A total of 427 human stool samples were collected during June-July 2016 in the municipality of Umbuzeiro and an overall prevalence of 3.04% (13/427) resulted positive by duplicate Kato-Katz thick smear. A total of 1,175 snails identified as *Biomphalaria straminea* were collected from 14 breeding sites along the Paraíba riverbank and distributed in 46 pools. DNA from human stool samples and pooled snails was extracted using the phenol/chloroform method. When performing the SmMIT-LAMP assay a total of 49/162 (30.24%) stool samples resulted positive, including 12/13 (92.31%) that were Kato-Katz positive and 37/149 (24.83%) previously Kato-Katz negative. By nested-PCR, only 1/46 pooled DNA snail samples was positive. By SmMIT-LAMP assay, the same sample also resulted positive and an additional one was positive from a different breeding site. Data of human and snail surveys were used to build risk maps of schistosomiasis incidence using kernel density analysis.

**Conclusions/Significance:**

This is the first study in which a LAMP assay was evaluated in both human stool and snail samples from a low-transmission schistosomiasis-endemic area. Our SmMIT-LAMP proved to be much more efficient in detection of *S*. *mansoni* in comparison to the 'gold standard' Kato-Katz method in human stool samples and the reference molecular nested-PCR in snails. The SmMIT-LAMP has demonstrated to be a useful molecular tool to identify potential foci of transmission in order to build risk maps of schistosomiasis.

## Author summary

In Brazil, around 1.8 million people, mostly in the northeastern region of the country, are thought to be infected with *Schistosoma mansoni*. Snails of the genus *Biomphalaria* serve as intermediate hosts of the *S*. *mansoni*. A special program for schistosomiasis control was implemented more than 40 years ago in Brazil, decreasing prevalence, morbidity, and mortality over the past years. PCR-based diagnostic methods have been successfully applied in a few endemic areas of schistosomiasis in Brazil, although they are not still widely used due to the highly technical requirements making them unviable for routine application in field conditions. Loop-mediated isothermal amplification (LAMP) technology could be a powerful tool to apply for point-of-care testing in resource-poor settings. In previous work, a LAMP-based method to detect *S*. *mansoni* DNA, called SmMIT-LAMP, was developed by our research group to detect *S*. *mansoni* DNA testing stool samples from experimentally infected mice. Here, with the aim to apply SmMIT-LAMP as a cost-effective molecular tool for the detection of *S*. *mansoni* in field applicable conditions, we assess SmMIT-LAMP in human and snail samples collected in an endemic area of Brazil. The results obtained by Kato-Katz analysis of human stool samples and nested-PCR performed in snails were compared with the SmMIT-LAMP assay. It is the first time that a LAMP-based method has been used to identify potential transmission foci and to evaluate the epidemiological risk of acquiring schistosomiasis.

## Introduction

Schistosomiasis has been a public health problem in Brazil for decades. Around 1.8 million people, mostly in the coastal states of Brazil, are thought to be infected with *Schistosoma mansoni* and 25 million living at risk of contracting the disease in America [1]. Nineteen of the twenty-six federal states of Brazil are affected by the disease, especially in the northeastern region of the country. A schistosomiasis control program was implemented more than 40 years ago, decreasing prevalence, morbidity, and mortality over the past years [2]. Nevertheless, parasitological or immunological tests are not effective for detecting *S*. *mansoni* infection in low prevalence areas although polymerase chain reaction (PCR)-based diagnostic methods have been successfully developed and applied in endemic areas of schistosomiasis in Brazil [3–7]. They are not widely used in low-income countries due to the highly technical requirements and need for skilled personnel, making them unviable for routine application in field conditions.

Snails of the genus *Biomphalaria* are well known for their role as intermediate hosts of *S*. *mansoni* which are able to produce hundreds or thousands of cercariae for months. Detection of cercarial shedding by infected snails after exposure of the specimens to light has traditionally been the method used to detect active sites for snail-to-human transmission [8]. This technique has several disadvantages: non-shedding of snail during the prepatent period, lack of experienced personnel for the identification of the acute infection, and difficulty in differentiating the morphology of the cercariae between trematodes species. To avoid these limitations, the detection of *S*. *mansoni* DNA in snail has been a good option offering greater sensitivity than classical methods with the advantage of detecting parasite of pooled snail samples. Therefore, several PCR-based assays have been developed to detect snails infected with *S*. *mansoni* [9, 10]. In Brazil, a nested-PCR for monitoring *S*. *mansoni*-infected *Biomphalaria* spp. has been used as the most common technique to identify active foci of schistosomiasis transmission [11, 12]. However, as noted, PCR-based techniques are difficult to apply in many endemic areas of schistosomiasis.

Loop-mediated isothermal amplification (LAMP) technology [13] is a powerful tool to apply for point-of-care testing in resource-poor settings because it is a rapid single-step assay which does not require a thermal cycler and results can be visualized by naked eye; additionally, LAMP technology can be used quantitatively using real-time assays, thus potentially providing information about level of infection [14]. LAMP assays have been developed for molecular detection and diagnostics of several Neglected Tropical Diseases (NTDs) and applied mainly in those produced by protozoa as human African trypanosomiasis and leishmaniasis [15, 16]. Additionally, LAMP assays have been successfully described for detecting NTDs produced by helminth parasites, including filariasis, soil-transmitted helminthiases and foodborne trematodiases [17–22]. Recently, several monitoring LAMP-based assays have also been developed for the detection of schistosomes [23–27]. In a previous work, a 620 bp sequence corresponding to a mitochondrial *S*. *mansoni* minisatellite DNA region was selected as a target for designing a LAMP-based method to detect *S*. *mansoni* DNA. This technique, called SmMIT-LAMP, was developed by our research group to detect *S*. *mansoni* DNA in stool samples from infected mice [28]. The specificity of this assay was determined against 16 DNA of different parasites -including several helminths and protozoa- and the limit of detection was established in 1 fg using *S*. *mansoni* DNA.

With the aim to apply SmMIT-LAMP as a cost-effective molecular tool for the detection of *S*. *mansoni* in field applicable conditions, in this study we assess SmMIT-LAMP in human and snail samples collected in an endemic area of Brazil. Moreover, the results obtained by Kato-Katz analysis of human stool samples and nested-PCR performed in snails have been compared with the SmMIT-LAMP assay. It is the first time that a LAMP-based method has been used to identify transmission foci and to evaluate the epidemiological risk of acquiring schistosomiasis.

## Methods

### Ethic statement

The study was approved by the Aggeu Magalhães Institute Ethics Committee (protocol approval no. CAAE 56338916.6.0000.5190). Volunteers were given detailed explanations about the aims, procedures and possible benefits of the study. Written informed consent was obtained from all subjects prior to the collection of biological samples for parasitological and molecular evaluation. Parents or guardians of children who participated in the study provided written informed consent on the child's behalf. All participants with confirmed infection received appropriate treatment. All samples were coded and treated anonymously. Procedures were performed in accordance with the ethical standards laid down in the Declaration of Helsinki as revised in 2013.

### Study area, population and mapping

**Fig 1:**
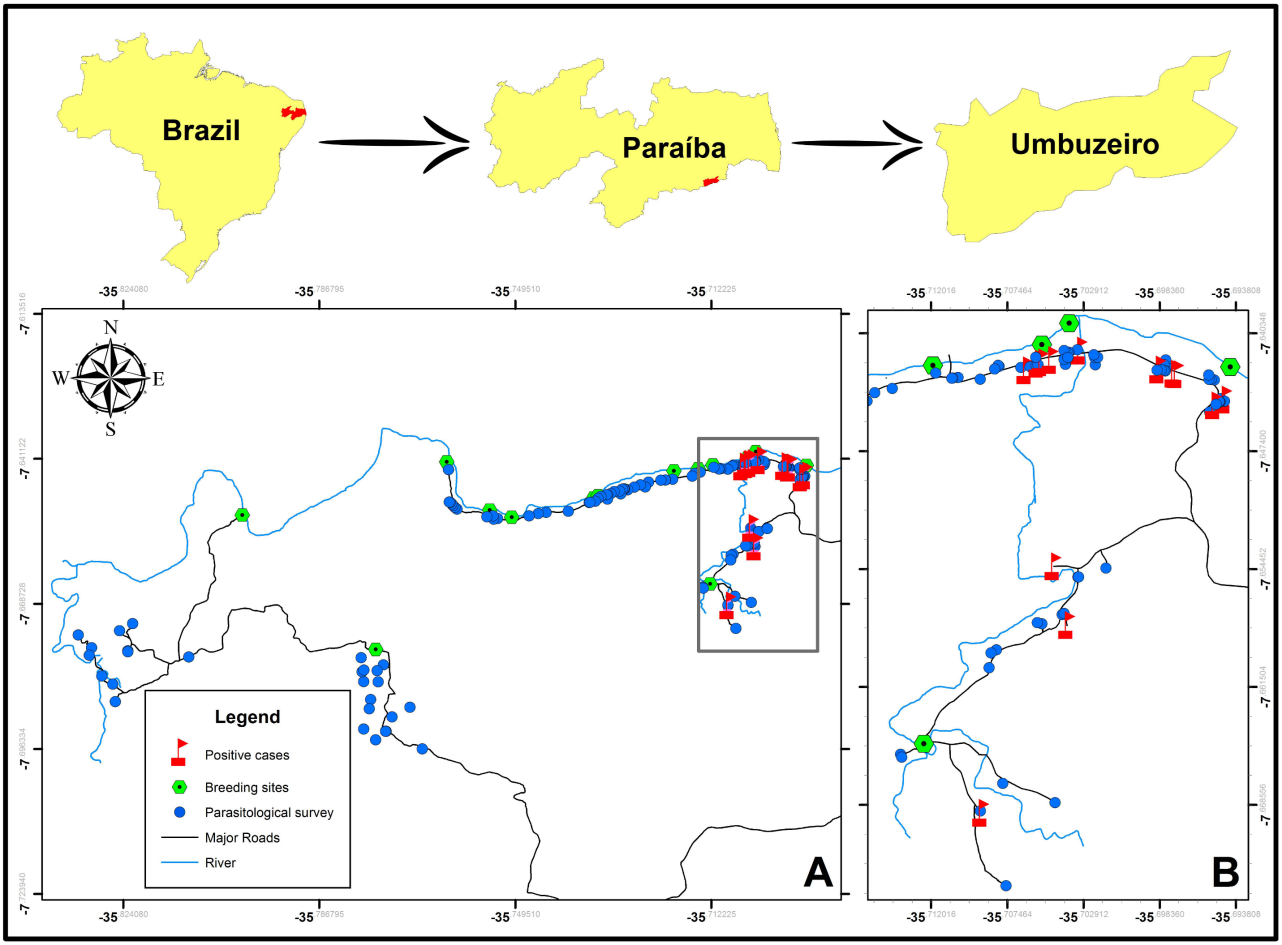
Map of the study area in the municipality of Umbuzeiro, located in the Agreste region of Paraíba State in the Northeast Region of Brazil. (A) Distribution of households included in parasitological survey (grey points); Kato-Katz positive samples (red flags) and breeding sites of *Biomphalaria straminea* (green points). (B) Enlargement of the area of the study where Kato-Katz positive results were obtained.

The study was conducted during June and July 2016 in the municipality of Umbuzeiro, located in the Agreste region of Paraíba State in the Northeast Region of Brazil (Fig 1). The municipality of Umbuzeiro covers an area of 181,327 km^2^ and has a population of 9,300 inhabitants (51.28 inhabitants/km^2^) with 3,986 and 5,314 people living in urban and rural areas, respectively, at 2010 census [29]. This location was chosen for the study because it is a known rural area with a low-endemicity for schistosomiasis and no records of mass treatment of the population within the last ten years. Moreover, this municipality is crossed by the Paraíba River, the only hydrographic basin in the region and the population has work and leisure activities centered on the river. The locality was georeferenced by means of the global positioning system (GPS) technology, using a GPS receiver (Garmin, model eTrex) with minimum accuracy of 10 meters, configured in the Universal Transverse Mercator (UTM) projection Datum SIRGAS 2000. Using the TrackMaker Pro software, the GPS receiver data was transferred to a computer, making it possible to save files (map, case distribution, breeding sites and foci) in format used in the spatial data analysis, which was done by means of the ArcGis software 10.1. The shapefiles of Brazil, Paraíba and Umbuzeiro, which is part of the base layers of the maps presented, as well as all the satellite images of Umbuzeiro in this work were generated using the sensor Sentinel 2 of the European Space Agency (ESA) (https://sentinel.esa.int/web/sentinel/user-guides/sentinel-2-msi) with Open Access CC-BY License (http://open.esa.int/). The satellites images were taken in June/2016.

### Collection of samples

#### Human stool sampling and parasitological tests

A total of 427 participants from 127 households were included in the study. The average household size was 3.36 people per household. Participants, including 199 males (46.60%) and 228 females (53.39%) with a median age of 29.81 (range 1–91; SD: 20.83) were registered and recruited from door to door for the parasitological survey. Of the total of 427 participants, 107 (25.05%) were children with the age range of 1–12 years old. Each participant was given a parasitological flask for stool collection. Samples were collected on a second visit the following morning. A single stool sample was individually obtained from each participant.

After collection, samples were transported to the Schistosomiasis Laboratory and Reference Service of Aggeu Magalhães Institute/Fiocruz for parasitological screening by Kato-Katz technique [30]. Duplicate Kato-Katz thick smear slides were prepared from each stool sample for the detection of *S*. *mansoni* eggs by well-trained technicians. After preparation of slides, the remaining samples were kept at -20°C until further DNA extraction for molecular analysis as described below.

#### Snail sampling and processing

To determine the snail collection locations a survey along the Paraíba riverbank was carried out according to the presence of *Biomphalaria* snail and the use of the river for leisure, labor activities or crossing path as the main epidemiological criteria. *Biomphalaria* snails were *in situ* sorted out based on shell characteristics. A total of 14 snail breeding sites were located and selected for the study. In each breeding site, snails were collected using scoops and tweezers for 15 min and placed into properly labeled moistened ventilated plastic tubs for later transportation to the Schistosomiasis Laboratory and Reference Service of Aggeu Magalhães Institute /Fiocruz.

A number of specimens from each breeding site were randomly selected for species confirmation using standard taxonomic identification keys [31–35]. In order to identify the snail breeding sites as potential transmission foci for schistosomiasis, all the snails were exposed to artificial light to shed the cercariae in case they were infected [36]. Then, snails were divided into 46 batches containing a maximum number of 30 snails/pool for easy handling and processing maintaining their identification according to the different 14 breeding sites. Afterwards, snails were storage at -20°C until further DNA extraction for molecular analysis.

### DNA purification and molecular analysis

#### DNA purification

Human stool samples (5–10 g/each) and pools of snails (4–30 speciments/batch) were used for DNA extraction using an adapted phenol/chloroform method [37]. Briefly, feces or whole snails -including the shell and the soft parts- were homogenized in 10 mL of lysis solution (10mM NaCl, 0.5% SDS, 25 mM EDTA, 10 mM Tris-HCl, pH 8.0). Following a brief centrifugation (5,000 rpm), the supernatant was extracted with phenol/chloroform and precipitated with isopropanol. The pellet was resuspended in 1 mL of TE buffer. Then, 2 μL of DNA purified from stool or snail pooled samples were used for molecular analysis.

#### Two-step nested PCR for snails analysis

A nested PCR was performed using two pairs of primers in two sequential reactions as previously described by Melo et al. [11] with modifications. Briefly, 50 pmol of outer primers (Schfo19 and Unvfo2) were used in the first PCR, and 50 pmols of internal primers (Schfo17 and Schre16) were used in the second PCR. Two microlitres of the product of the first PCR were used as template for the second PCR. The mixtures for both PCR reactions, were prepared containing 10 mM Tris-HCl, 50 mM KCl, 1.5 mM MgCl_2_, 0.2 mM dNTP 50 pmol of each primer and 2.5 units of Taq DNA polymerase (Amersham Pharmacia Biotech, USA). In the first reaction, program was run for 30 cycles, consisting of denaturation at 92°C for 30 s, annealing at 65°C for 1 min and extension at 72°C for 1 min. In the second PCR, program was the same, with the exception of annealing temperature at 58°C. Several positive (*S*. *mansoni* DNA) and negative (no template) controls were included in each PCR run. The PCR products (5 μL) were visualized in 2% agarose gels and photographed using a UV light system.

### SmMIT-LAMP for human stool samples and snails analysis

Of the total of 427 human stool samples included in the study, only 162 samples (collected in a zone at the northeast of the study area where Kato-Katz positive samples were detected) were tested using the reaction mixture and specific primer set for LAMP assay -SmMIT-LAMP- previously established by Fernández-Soto et al. [28]. All pooled snail samples were also analyzed using the same LAMP assay. The SmMIT-LAMP method amplifies a specific sequence corresponding to a mitochondrial *S*. *mansoni* minisatellite DNA region (GenBank Acc. No. L27240). Briefly, the reaction was carried out in 25 μL reaction mixture containing 40 pmol each of FIP and BIP primers, 5 pmol of each F3 and B3 primers, 1.4 mM of each dNTP (Intron), 1x Isothermal Amplification Buffer -20 mM Tris-HCl (pH 8.8), 50 mM KCl, 10 mM (NH_4_)_2_SO_4_, 2 mM MgSO_4_, 0.1% Tween20- (New England Biolabs, UK), 1 M betaine (Sigma, USA), supplementary 6 mM of MgSO_4_ (New England Biolabs, UK) and 8 U of *Bst* 2.0 WarmStart DNA polymerase (New England Biolabs, UK) with 2 μL of template DNA. Reaction tubes were placed in a heating block at a constant temperature of 63°C for 60 min and then heated at 80°C for 5 min to stop the reaction. In all SmMIT-LAMP trials positive *(S*. *mansoni* DNA) and negative (water) controls were always included.

The LAMP-positive results could be visually inspected by naked eye by color change after adding 2 μL of 1:10 diluted 10,000x concentration fluorescent dye SYBR Green I to the reactions tubes. Green fluorescence was clearly observed in successful LAMP reaction, whereas it remained the original orange in the negative reaction. To avoid as much as possible the potential risk of cross-contamination with amplified products, all tubes were briefly centrifuged and carefully opened before adding the fluorescent dye.

### Spatial data analysis

Data of human and snail surveys and results of parasitological and molecular analysis were used to build risk maps. Based on the number of snails collected in each station a thematic map demonstrating the abundance of snails in the breeding sites and foci of transmission was built. A kernel density analysis was also performed to draw a risk map of schistosomiasis incidence according to the diagnostic methods used for detection. Kernel Density Estimation (KDE) is a statistical technique of interpolation, nonparametric method, which produces a continuous surface (cluster) of the density calculated at all locations for visual identification of hotspots without changing their local characteristics [38, 39]. The area unit was defined in m^2^ and the kernel spatial resolution in 10 meters.

### Statistical methods

Statistical analyses were performed using GraphPad Prism software package (version 6, GraphPad Software, Inc., San Diego, CA, USA; https://www.graphpad.com). Comparison of LAMP results with those obtained by microscopy were analyzed by McNemar's test for matched pairs. Comparisons were considered significant at a p-value < 0.05. The diagnostic sensitivity, specificity, positive predictive value (PPV) and negative predictive value (NPV) were calculated for the SmMIT-LAMP and the Kato-Katz method using the MedCalc statistical program version 15.2.2 (MedCalc Software, Ostende, Belgium) according to the software instruction manual (www.medcalc.org).

## Results

### Parasitological analysis by Kato-Katz

A total of 13/427 (3.04%) human stool samples were positive by duplicate Kato-Katz thick smears, including samples obtained from 5 males and 8 females (median age 45; range 14–90; SD 22.76). In all Kato-Katz positive slides, the *S*. *mansoni* egg counts was very low, as well as the number of egg per gram of feces (EPG) with an average from 12 to 180 (Table 1). Up to 4/13 slides resulted negative in one of the two analyses.

**Table 1:** *Schistosoma mansoni* eggs count in human stool samples detected by duplicate Kato-Katz thick smears. The gender and age of the patients are also indicated.

Sample Number	Gender	Age	KKT—1	KKT—2	EPG
1	F	14	3	3	72
2	M	65	0	1	12
3	F	34	1	2	36
4	F	63	4	8	144
5	F	90	6	7	156
6	M	26	1	1	24
7	M	32	9	6	180
8	F	20	1	0	12
9	F	57	1	1	24
10	F	20	1	0	12
11	M	64	0	1	12
12	M	45	2	1	36
13	F	55	5	1	72

KKT-1, first Kato-Katz technique; KKT-2, second Kato-Katz technique; EPG, eggs per gram of feces for each sample.

The spatial distribution of parasitological positive cases is represented in Fig 1A. Of the total of 427 participants in the study delivering their stool samples, all Kato-Katz positive cases were detected in a zone located at the northeast of the study area (Fig 1B). In that zone, a total of 162 samples having tested previously collected in the parasitological survey, counting the 13 Kato-Katz positive samples and 149 Kato-Katz negative samples. These 162 samples were further subjected to molecular analysis by LAMP assay as described below.

### SmMIT-LAMP analysis of human stool samples

**Fig 2:**
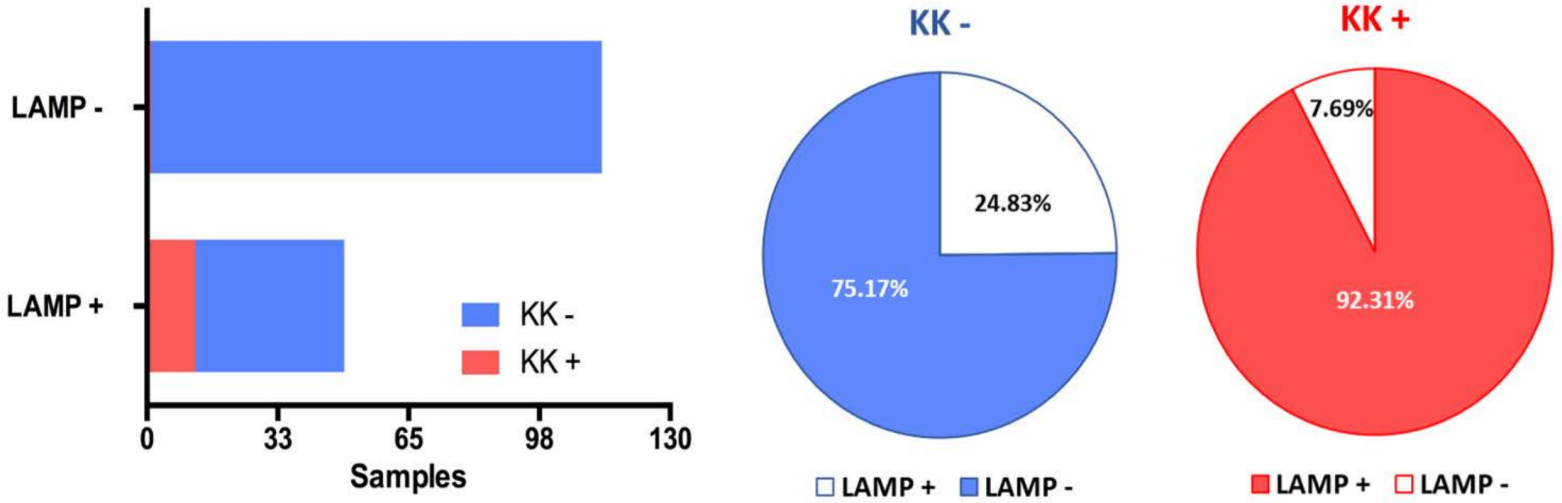
Overall results obtained by LAMP assay in comparison to Kato-Katz. Blue and red color shows negative (KK-) and positive (KK+) samples by Kato-Katz. Bars represent the number of LAMP positive (LAMP+) and negative (LAMP-) samples. Sectors show the percentage of samples LAMP positive and negative in comparison to Kato-Katz positive and negative samples.

**Fig 3:**
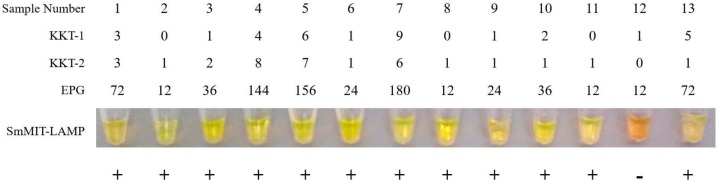
Results obtained by SmMIT-LAMP in comparison to Kato-Katz positive samples for *Schistosoma mansoni*. Sample Number, samples 1–13 positive by Kato-Katz; KKT-1, first Kato-Katz test; KKT-2, second Kato-Katz test; EPG, eggs per gram of feces for each sample; SmMIT-LAMP, visual inspection of reaction tubes positive (green, +) and negative (orange, -), respectively.

The SmMIT-LAMP overall results obtained after testing the stool samples in comparison to Kato-Katz results are showed in Fig 2. When performing the SmMIT-LAMP assay a total of 49/162 (30.24%) stool samples resulted positive, including 12 of the 13 (92.31%) previously resulting Kato-Katz positive and, additionally, 37/149 (24.83%) previously Kato-Katz negative. The SmMIT-LAMP results for human stool samples resulting Kato-Katz positive are shown in Fig 3. Despite no child had been positive for *S*. *mansoni* by the Kato-Katz method, up to 12 children under 12 years old were positive for *S*. *mansoni* using the LAMP assay, which represent 24.5% of the total 49 positive cases detected by the molecular technique.

MacNemar's test showed a statistically significant relationship between LAMP results and microscopy-detected *S*. *mansoni* infections (p-value<0.0001). Considering the microscopy findings by Kato-Katz as the reference standard, the following diagnostic parameters were calculated for the SmMIT-LAMP in this study: 92.86% sensitivity (95% CI: 66.13% -99.82%); 80.11% specificity (95% CI: 73.64% -85.59%); 26.00% positive predicted value (95% CI: 20.28% -32.67%) and 99.33% negative predicted value (95% CI: 95.75% -99.90%).

### Snail identification, cercarial shedding, nested PCR and SmMIT-LAMP analysis

A total of 1,175 snails were collected with an average number of specimens per breeding site of 83.92 (range 4–370; SD: 109.44). All snails were identified as *Biomphalaria straminea*. None of the snails examined by exposure to artificial light for cercariae to emerge was infected.

**Fig 4:**
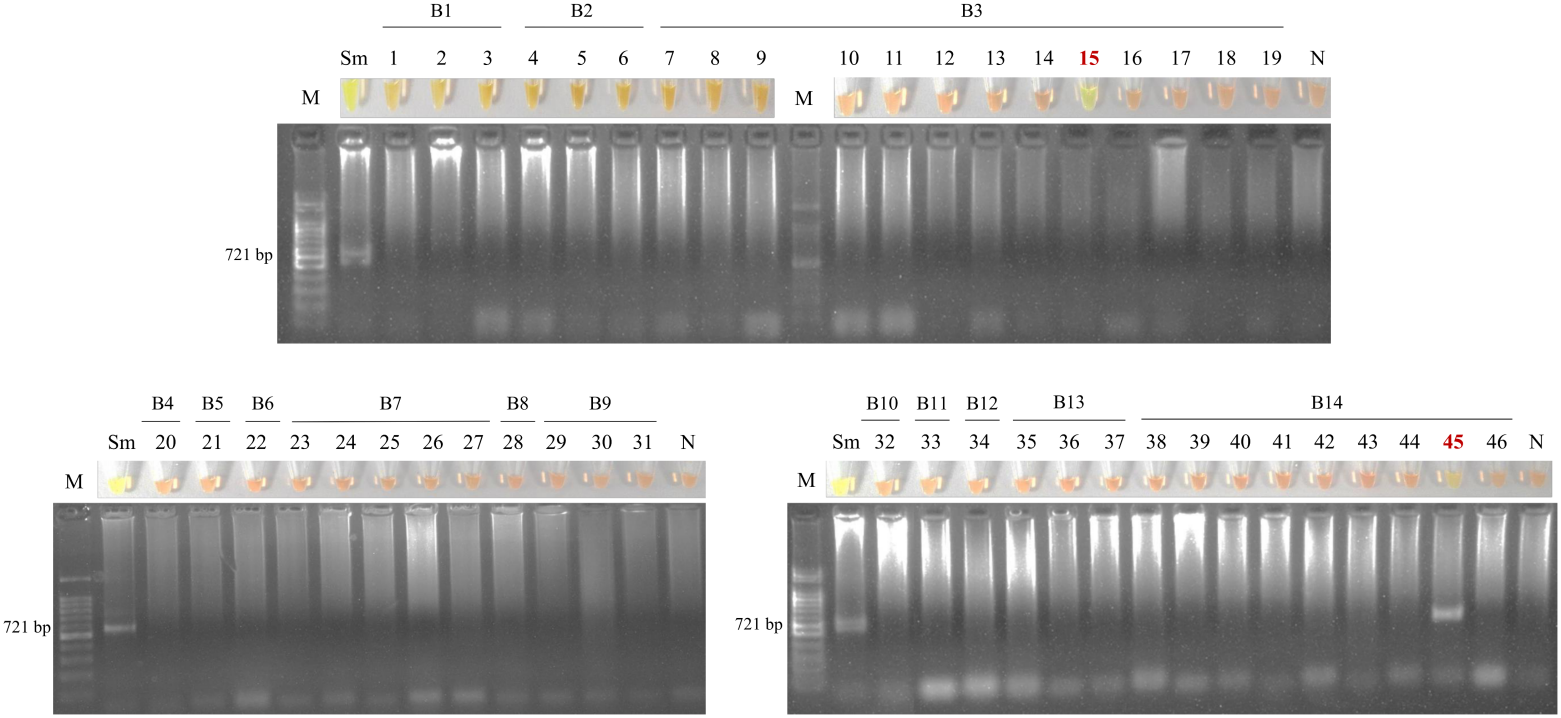
Examination of the 46 pooled snail samples by SmMIT-LAMP and nested-PCR. Figure shows the tubes with LAMP results (up) and electrophoresis with nested-PCR results (down). B1-14, the 14 breeding sites where snails were collected; lanes 1–46, pooled snail samples; lanes M, molecular weight marker; lanes Sm: positive control (*S*. *mansoni* DNA); lanes N, negative control (no DNA template). Green fluorescence was observed in two pooled snail samples: no.15 and no. 45 (in which a nested-PCR positive result was also obtained) from the breading sites B3 and B14, respectively.

**Fig 5:**
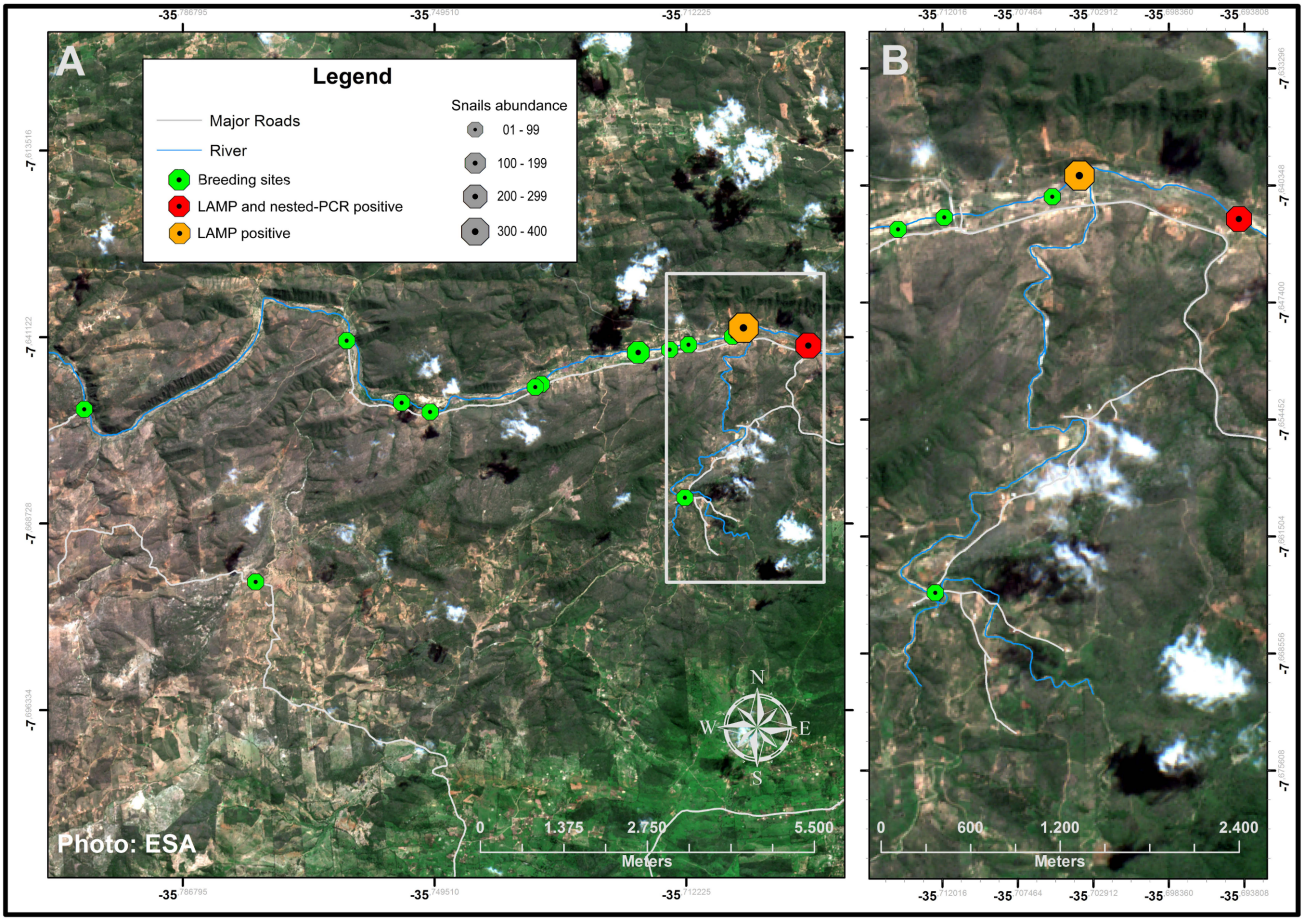
Geographical distribution of breeding sites showing positive pooled snails samples by molecular assays. (A) Breeding sites and identification of potential foci of transmission of *S*. *mansoni* either by using LAMP and nested-PCR (red point), or by LAMP assay alone (orange point). The abundance of snails is also indicated (circle diameter size). (B) Enlargement of the area of the study where positive cases for *S*. *mansoni* were identified.

Testing the 46 pooled snail DNA samples by nested-PCR, only one resulted positive (no. 45). By SmMIT-LAMP assay, the same pooled snail sample also resulted positive and another pool (no. 15) was positive from a different breeding site. The SmMIT-LAMP and nested-PCR results are shown in Fig 4 and the geographical distribution of breeding sites where pooled snails samples resulted positive by molecular assays is shown in Fig 5. In these two points, the abundance of snails was the largest of the survey. Additionally, the two-pooled snail positive samples were located in the same area where the highest number of patients with microscopy-positive results were detected. Thus, two potential foci of schistosomiasis transmission were identified in the study area.

### Risk of schistosomiasis infection

**Fig 6:**
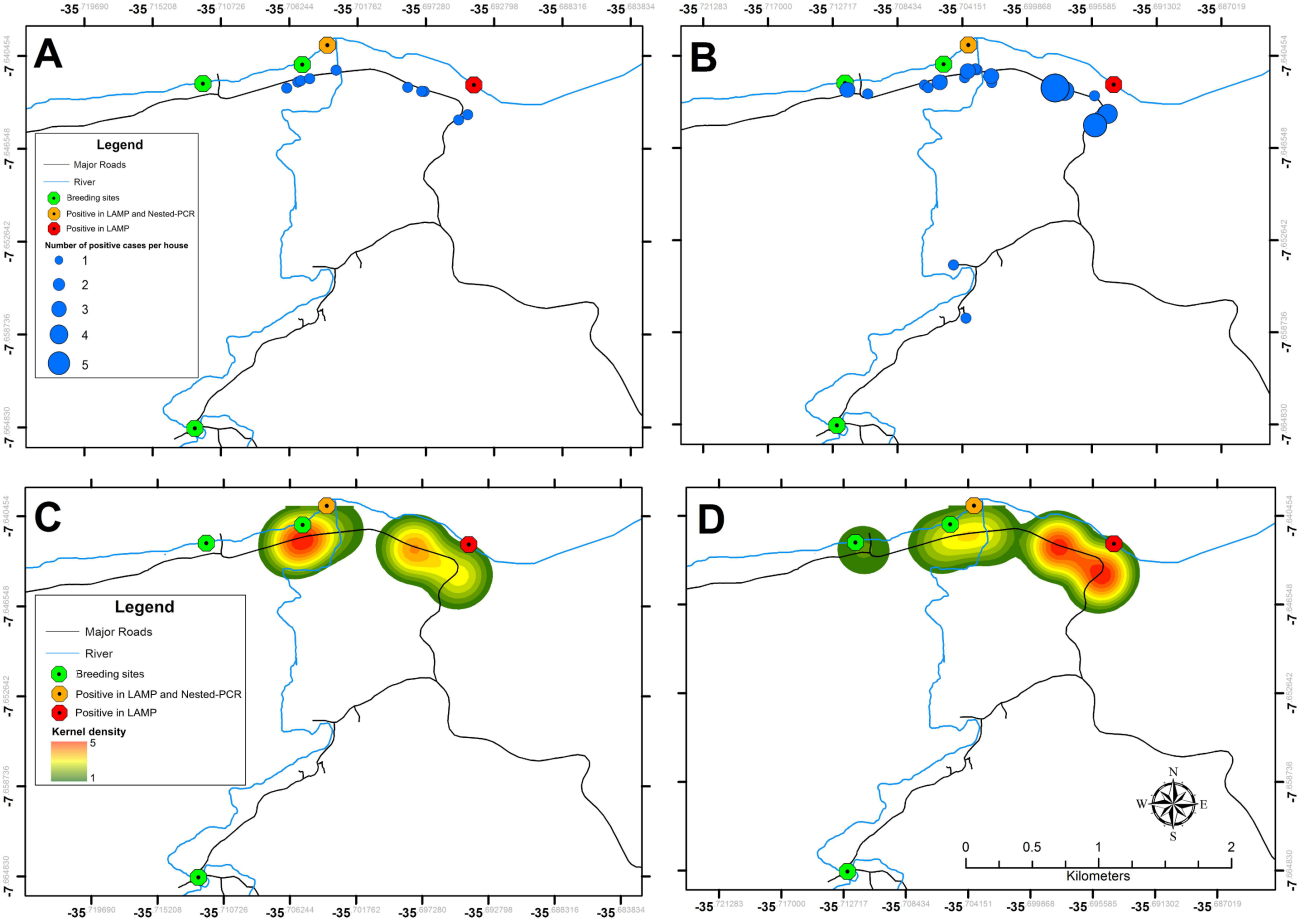
Risk maps of the schistosomiasis incidence according to Kato-Katz and SmMIT-LAMP results. (A) Distribution of cases by households including in the parasitological survey using Kato-Katz. (B) Distribution of cases by household using LAMP assay. (C) Kernel risk map of the occurrence of cases by Kato-Katz method. (D) Kernel risk map of the occurrence of cases by the LAMP assay.

The distribution of positive cases by both microscopy and SmMIT-LAMP in households included in the study, as well as risk maps of schistosomiasis infection generated with the Kernel density method are shown in Fig 6. Only one positive result per household was detected when using the Kato-Katz technique (Fig 5A), whereas up to five positive results per household could be obtained when using the SmMIT-LAMP assay (Fig 5B). The maps of potential risk areas of schistosomiasis transmission based on this study, either by Kato-Katz tests (Fig 5C), and SmMIT-LAMP (Fig 5D) are shown. Two foci of schistosomiasis transmission were located at the breeding sites where pooled snail samples resulted positive by molecular assays.

## Discussion

Our study was conducted in a known low prevalence area of schistosomiasis in Brazil. Kato-Katz results obtained in the population survey corroborated previous results published from the study area [40]. Only thirteen stool samples (3.04%) resulted positive by microscopic analysis, including up to ten samples with light infections (1–99 EPG), where four of those thirteen did not show the presence of *S*. *mansoni* eggs in one of the two slides examined. These data are in line with the known low sensitivity of the Kato-Katz technique for diagnosing schistosomiasis in areas of low prevalence and parasite load [4, 41]. Molecular assays arise as a potential alternative to traditional parasitological methods in situations were highly sensitive diagnostic test are needed [42]. In this context, we tried to evaluate our SmMIT-LAMP assay in an area where the Kato-Katz previously showed low sensitivity. When performing the SmMIT-LAMP, the number of positive samples detected increased up to 49, with an overall prevalence of 30.24%. Moreover, from the number of samples positive by Kato-Katz, up to 92.31% were LAMP-positive, thus indicating a higher performance of the technique. Thus, our SmMIT-LAMP seems to be much more sensitive than microscopic detection of eggs, commonly used as the classical reference test for intestinal schistosomiasis [28].

We assume that LAMP positive results are *S*. *mansoni*-infected patients considering the known low sensitivity of the microscopy and the high sensitivity and specificity of the molecular assay. In a previous work of our group, the SmMIT-LAMP was developed and the specificity of the assay was tested against more than 20 DNA of different parasites, including helminths and several protozoa. The specificity of the mitochondrial DNA sequence used for primer design was also confirmed using different bionformatic tools [28]. Additionally, negative controls were always included in all LAMP assays in order to detect potential contaminations with *S*. *mansoni* DNA. If any negative control used in the trials showed DNA amplification, the result was invalidated and the reaction was discarded and repeated. Thus, we are confident that the LAMP-positive results could only be due to the presence of *S*. *mansoni* DNA in the stool samples. Other studies evaluating molecular assays versus microscopy have reported a similar increment of positive cases [42], thus representing a better performance of the molecular assays in comparison with parasitological methods. One KK-positive sample was LAMP-negative, resulting in a sensitivity of 92.86%. This sample presented absence of *S*. *mansoni* eggs in one of the two slides microscopically examined. This sensitivity is in accordance with other molecular assays for the diagnosis of *S*. *mansoni* [42], so the percentage of false negative is entirely aceptable.

Furthermore, *S*. *mansoni* DNA was extracted using a cost-effective phenol/cloroform method without compromising the sensitivity of the LAMP analysis. In this context, a number of LAMP assays have been previously reported for parasites detection with a minimal or no DNA extraction requirement, including schistosomes [25, 43, 44]. Moreover, the SmMIT-LAMP results are easily visualized by color change by naked eye. This is a great advantage for epidemiological surveys in low-income areas compared to other DNA-based molecular methods.

*Biomphalaria straminea* was the sole species identified as intermediate host in the study area. This finding is in accordance with a previous malacological survey in this region [45]. Among the three species of host snails for *S*. *mansoni* in Brazil, *B*. *glabrata* is considered to be the most epidemiologically important species since its geographical distribution overlaps with the distribution of schistosomiasis in Brasil. However, *B*. *straminea* is better adapted to all climatic variations and ecological conditions in Brazil [46]. Besides, *B*. *straminea* is considered more resistant to *S*. *mansoni* infection than other snail species [47]. Different epidemiological studies have demonstrated the utility of the nested-PCR method for *S*. *mansoni* detection in pooled *B*. *straminea* samples when the parasitological assays are not effective [11, 45]. In our work, one pooled sample of *B*. *straminea* was detected using this nested-PCR assay as reference, although no one was positive by classical cercarial shedding tests.

In order to test the SmMIT-LAMP in detecting *S*. *mansoni* in *B*. *straminea*, we analyzed the pooled snail samples and compared results with nested-PCR assay. The SmMIT-LAMP assay was originally designing on a sequence corresponding to a specific mitochondrial *S*. *mansoni* minisatellite DNA region [48]. This sequence was also previously used for designed a specific PCR-based method for detection of *S*. *mansoni* with no cross-reaction with other Brazilian trematodes which have snails of genus *Biomphalaria* as intermediate hosts [10]. When applying the two molecular methods for snail samples screening, two breeding sites wereidentified as potential foci of schistosomiasis transmission, one detected by both nested-PCR and SmMIT-LAMP and an additional one by SmMIT-LAMP. These two foci of transmission are located in the same study area where the Kato-Katz positive human cases were detected. In recent years, it has been reported the use of LAMP in large-scale screening of pooled field-collected snails for analyzing the transmission of schistosomiasis, as a simple and efficient tool for snails’ surveillance, including *S*. *mansoni* in Brazil [49, 27]. In our study, the SmMIT-LAMP assay was applied for the first time to evaluate the *S*. *mansoni* infection not only in pooled field-collected snails but also in human stool samples.

Data obtained in both SmMIT-LAMP and Kato-Katz tests were used to create Kernel density-based maps of risk of schistosomiasis. The Kernel density has previously been used to build maps of risk for several helminthiases, including schistosomiasis [50–52]. The risk areas obtained mapped close to the snail breeding sites identified as foci of schistosomiasis transmission by the SmMIT-LAMP and nested-PCR. In those breeding sites the highest contact between the population and the river was observed for work activities (extraction of sand from the river), domestic activities (washing clothes and dishes), and leisure activities (fishing and children's recreation). All these activities are known to be associated with transmission of schistosomiasis [53, 54]. In addition, this area is commonly used by the inhabitants as a route for crossing the river further increasing the risk of infection.

In summary, this is the first study in which a LAMP assay was evaluated in both human stool and snail samples from a low-transmission schistosomiasis-endemic area. Our SmMIT-LAMP proved to be much more efficient in detecting *S*. *mansoni* in comparison to the 'gold standard' method (Kato-Katz) in human stool samples and the reference molecular nested-PCR in snails. Moreover, SmMIT-LAMP has demonstrated to be a useful molecular tool to identify foci of transmission in order to build risk maps of schistosomiasis.

## Supporting information

S1 Checklist STARD checklist. (DOCX)

S2 Checklist Flowchart. (PDF)

S3 Checklist STROBE checklist. (DOCX)
