# Supplementary figures and images for: A field survey using LAMP assay for detection of Schistosoma mansoni in a low-transmission area of schistosomiasis in Umbuzeiro, Brazil: Assessment in human and snail samples
Source: PLoS Negl Trop Dis. 2018 Mar 13;12(3):e0006314. doi: 10.1371/journal.pntd.0006314 (PMC5849311; doi:10.1371/journal.pntd.0006314)

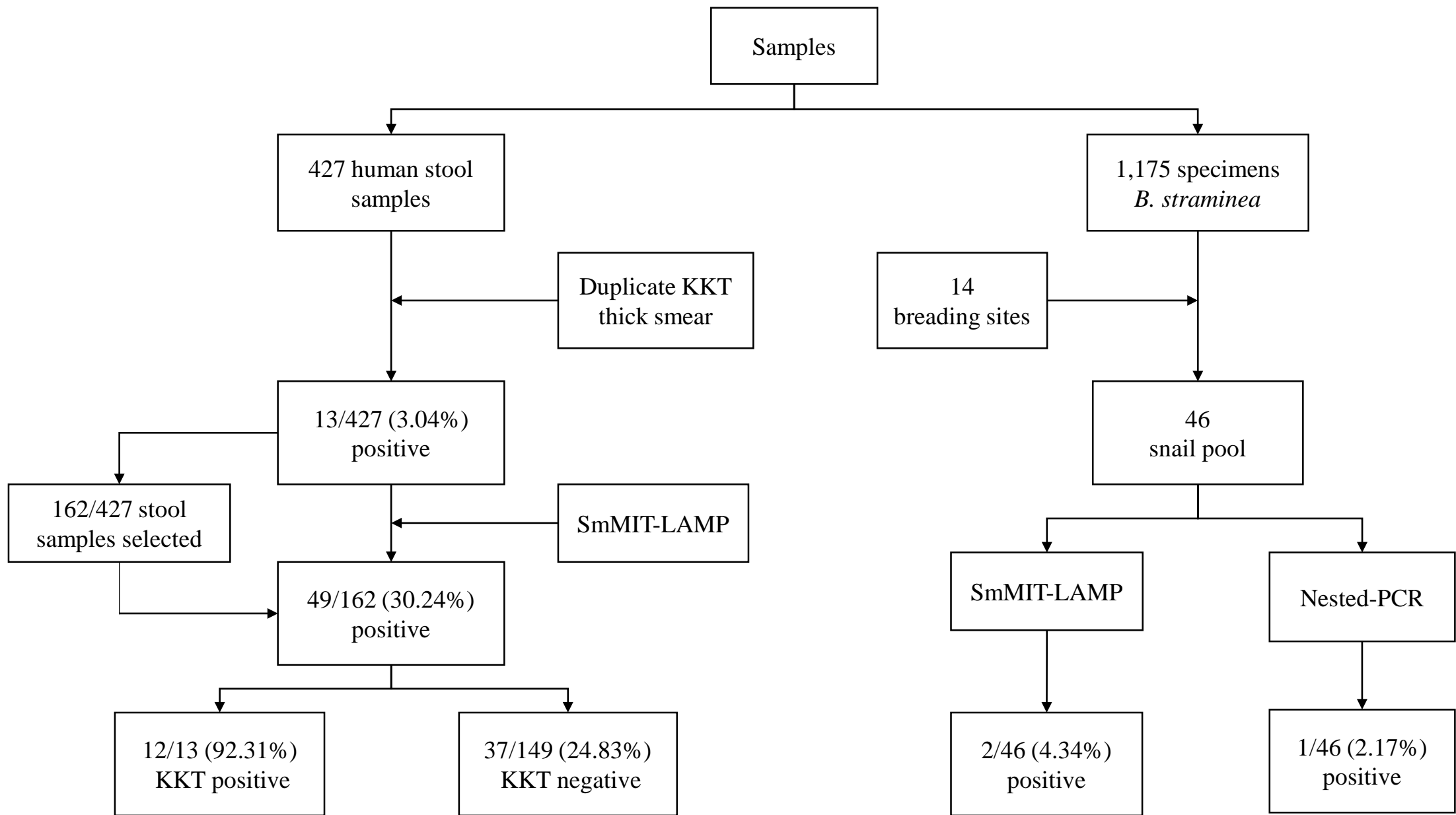

Supplement: S2 Checklist — (PDF) [file pntd.0006314.s002.pdf]
